# Hydroxyurea Scavenges Free Radicals and Induces the Expression of Antioxidant Genes in Human Cell Cultures Treated With Hemin

**DOI:** 10.3389/fimmu.2020.01488

**Published:** 2020-07-17

**Authors:** Sânzio Silva Santana, Thassila Nogueira Pitanga, Jeanne Machado de Santana, Dalila Lucíola Zanette, Jamile de Jesus Vieira, Sètondji Cocou Modeste Alexandre Yahouédéhou, Corynne Stéphanie Ahouefa Adanho, Sayonara de Melo Viana, Nivea Farias Luz, Valeria Matos Borges, Marilda Souza Goncalves

**Affiliations:** ^1^Instituto Gonçalo Moniz, Fundação Oswaldo Cruz (IGM/FIOCRUZ-BA), Salvador, Brazil; ^2^Faculdade de Biomedicina, Universidade Católica do Salvador (UCSal), Salvador, Brazil; ^3^Faculdade de Farmácia, Universidade Federal da Bahia (UFBA), Salvador, Brazil

**Keywords:** sickle cell anemia, hydroxyurea, hemin, antioxidant response, Nrf2

## Abstract

The excessive release of heme during hemolysis contributes to the severity of sickle cell anemia (SCA) by exacerbating hemoglobin S (HbS) autoxidation, inflammation and systemic tissue damage. The present study investigated the effect of hydroxyurea (HU) on free radical neutralization and its stimulation of antioxidant genes in human peripheral blood mononuclear cells (PBMC) and human umbilical vein endothelial cells (HUVEC) in the presence or absence of hemin. HU (100 and 200 μM) significantly reduced the production of intracellular reactive oxygen species (ROS) induced by hemin at 70 μM in HUVEC. HUVECs treated with HU+hemin presented significant increases in nitric oxide (NO) production in culture supernatants. HU alone or in combination with hemin promoted the induction of superoxide dismutase-1 (*SOD1*) and glutathione disulfide-reductase (*GSR*) in HUVECs and PBMCs, and glutathione peroxidase (*GPX1*) in PBMCs. Microarray analysis performed in HUVECs indicated that HU induces increased expression of genes involved in the antioxidant response system: *SOD2, GSR*, microsomal glutathione S-transferase (*MGST1*), glutathione S-transferase mu 2 (*GSTM2*), carbonyl reductase 1 (*CBR1*) and klotho B (*KLB*). Significant increases in expression were observed in genes with kinase activity: protein kinase C beta (*PRKCB*), zeta (*PRKCZ*) and phosphatidylinositol-4-phosphate 3-kinase catalytic subunit type 2 beta (*PIK3C2B*). HU also induced a significant increase in expression of the gene p62/sequestosome (*p62/SQSTM1*) and a significant decrease in the expression of the transcriptional factor *BACH1* in HUVECs. Upstream analysis predicted the activation of Jun, miR-155-5p and mir-141-3p. These results suggest that HU directly scavenges free radicals and induces the expression of antioxidant genes via induction of the Nrf2 signaling pathway.

## Introduction

Hydroxyurea (HU) is a hydroxylated analog of urea, which was initially identified as a myelosuppressive drug that acts by inhibiting ribonucleotide reductase. After determining its antisickling effect, HU was approved in 1998 by the U.S. Food and Drug Administration (FDA) for the treatment of sickle cell anemia (SCA). SCA is a hereditary autosomal recessive disease, characterized by the homozygosity of the beta S (β^S^) allele (HbSS), which is derived from the GAG>GTG mutation in the sixth position of the β globin gene (*HBB*) (1). The pathophysiological condition of SCA is recurrent and characterized by a large production of reactive oxygen species (ROS) and reactive nitrogen species (RNS), which play a crucial role in the maintenance of inflammation (2–6).

The imbalance caused by increased oxidation-reduction (redox) reactions in the vascular microenvironment in SCA provokes important deleterious effects (4). Indeed, patients with SCA can present (i) intravascular and extravascular hemolysis with free heme release; (ii) autoxidation of HbS (3, 7); (iii) nitric oxide (NO) depletion and endothelial dysfunction (8, 9); (iv) ischemia-reperfusion events (10); (v) marked leukocyte dysfunction, conferring a non-effector response against pathogens, and the dysregulation of inflammatory equilibrium that increases susceptibility to secondary infections (11–13).

Despite the recent approval of L-arginine by the FDA, HU remains the drug most indicated for SCA patients who present a severe clinical profile (14, 15). Experimental studies have demonstrated that after oral administration, HU is absorbed, converted into a nitroxide radical and transported to the active site of the M2 subunit of the ribonucleotide reductase protein, inactivating the enzyme and generating cytotoxic suppression, most likely via the induction of an antioxidant response (16). Ware (17) pointed out the main benefits of HU therapy in patients with SCA: HU induces fetal hemoglobin (HbF) production through the activation of guanylate cyclase and reduces neutrophil and reticulocyte counts by inhibiting ribonucleotide reductase activity and bone marrow toxicity. Moreover, it decreases adhesiveness and improves the rheology of circulating neutrophils and reticulocytes, reduces hemolysis and improves erythrocyte hydration, promotes macrocytosis, reduces intracellular sickling and stimulates the release of NO as a potential local vasodilator. Despite these benefits, relatively few studies have specifically focused on the action of HU in alternative mechanisms that broaden the field of knowledge regarding its action and systemic effects.

We hypothesized that HU can act by decreasing ROS/RNS and stimulating antioxidant defense systems in endothelial cells and leukocytes. To this end, we investigated the effects of HU in human peripheral blood mononuclear cells (PBMC) and umbilical cord vein endothelial cells (HUVEC) pre-treated or not with hemin, an important pro-oxidant molecule released during hemolysis (3, 18, 19). We then specifically investigated the antioxidant effect of HU, as well as the expression of antioxidant genes, such as heme oxygenase-1 (*HMOX1*), superoxide dismutase-1 (*SOD1*), glutathione disulfide-reductase (*GSR*) and glutathione peroxidase (*GPX1*).

## Methods

### Drugs

HU, butylated hydroxytoluene (BHT) and L-ascorbate were purchased from Sigma Aldrich (St. Louis, MO, USA) and prepared following the manufacturer's instructions. After complete solubilization, drugs were sterilized by filtration using a 0.22 μm polyethersulfone membrane (PES) (Jet Biofil, Guangzhou, China) for use in culturing assays.

### Preparation of Hemin

Hemin (Sigma Aldrich, St. Louis, MO, USA), a ferric chloride hemin, was prepared from a 5 mM stock solution solubilized in 0.1 M NaOH using non-pyrogenic water under dark conditions. The hemin solution was then diluted in RPMI 1640 medium (Gibco, New York, NY, USA) to obtain optimal concentrations. Finally, a non-pyrogenic hemin solution was obtained following 0.22 μm PES-membrane filtration (Jet Biofil, Guangzhou, China) for use in cell culture assays.

### Scavenging Activity Assay of 2,2-Diphenyl-1-Picrylhydrazyl (DPPH)

DPPH free scavenging activity was assessed by a modified microplate assay method previously described by Li et al. (20). Initially, 200 μM of DPPH stock solution (Sigma Aldrich, St. Louis, MO, USA) was prepared in methanol p.a. (Synth, Diadema, SP, Brazil) 10–15 min before experimentation, stored in a sealed bottle, and kept away from light. For this assay, stock drug solutions were prepared, using methanol, at concentrations ranging from 3.13 to 800 μM/well. HU, as well as the antioxidant external controls BHT and L-ascorbate, were incubated for 30 or 60 min at a volume of 0.1 mL on 96-well flat-bottom microtiter plates (Greiner Bio-one, Monroe, North Carolina, USA) at a ratio of 1:1 (v/v), with the addition of DPPH (100 μM/well). All plates were covered and kept in the dark to minimize evaporation and to avoid the photosensitization of DPPH radicals. Finally, the plated solutions were homogenized for 5 sec, and absorbance was measured on a microplate reader (SpectraMax 190, Molecular Devices Corporation, Sunnyvale, CA) using Softmax software v. 5.0 (Molecular Devices, Sunnyvale, CA, USA) at a wavelength of 517 nm. DPPH radical scavenging activity was determined using the following equation: *Scavenging activity of DPPH (%)* = *[(Abs*_dpph_*-Abs*_drug_*)* × *100]/Abs*_dpph_.

### Cell Cultures

HUVECs were cultured in 25 cm^2^ cell culture flasks (Costar, Corning, NY, USA) containing 5 mL RPMI 1640 medium (Gibco, New York, NY, USA) supplemented with 10% heat-inactivated fetal bovine serum (FBS) (Gibco, New York, NY, USA), 20 mM glutamine (Sigma Aldrich, St. Louis, MO, USA), 10 mM HEPES, 5 mM NaOH and the following antibiotics: 100 U/mL penicillin and 10 mg/mL streptomycin (Sigma Aldrich, St. Louis, MO, USA). For all assays, HUVECs were used in passages 1–5 and phenotypically characterized by the evaluation of typical cobblestone morphology and surface tissue factor (CD142) (Supplementary Figure 1).

Human peripheral venous blood samples were collected from healthy volunteers (HbAA genotype) to obtain PBMCs. Written informed consent was obtained from all study participants, and the present protocol was conducted in compliance with the 1975 Helsinki Declaration and its amendments, as well as the Brazilian ethical guidelines (466-CNS-2012). PBMCs were obtained by Ficoll-Paque Plus (GE Healthcare, Uppsala, Sweden) density gradient centrifugation following the manufacturer's instructions. Both HUVEC and PBMC were cultivated in a humidified atmosphere at 37°C under 5% CO_2_.

### Cytotoxicity Assays

The cytotoxic effects of the drugs and hemin on HUVEC were assessed using a resazurin sodium salt reduction colorimetric assay. For this, 2 × 10^4^ cells/well (0.2 mL) were plated on 96-well plates (Costar, Corning, NY, USA) and cultivated for 20–24 h under the culture conditions described above, until reaching a confluency of 70–80%. Cells were then treated with HU in combination or not with hemin for 24 h. After incubation, the medium was collected and the wells were gently washed once with preheated (37°C) 0.85% saline solution to avoid cell damage and detachment. Finally, 0.1 mL of 12.5 μM resazurin sodium salt solution (Sigma Aldrich, St. Louis, MO, USA) diluted in RPMI 1640 with 10% FBS was added to each well, followed by incubation at 37°C under 5% CO_2_ in a humidified atmosphere for 3 h according to standardization protocols (Supplementary Figure 2A). Absorbance was simultaneously read at wavelengths of 570 and 600 nm on a microplate reader. Cell viability was determined by measuring the percentage of sodium salt (deep blue fluorescent compound) that was reduced to resorufin (pink fluorescent product). For PBMC cytotoxicity assays, 3 × 10^5^ cells were incubated for 24 h with HU in combination or not with hemin. Cytotoxicity was assessed using propidium iodide (BD, Pharmigen, USA) following the manufacturer's specifications. For each sample, 20,000 events were acquired on a BD LSRFortessa™ cytometer (Biosciences, San Jose, CA, USA).

### Determination of Intracellular ROS

The detection of reactive oxygen species was determined in HUVECs using a 2′, 7′-dichlorodihydrofluorescein diacetate (DCFH-DA) probe (Sigma Aldrich, St. Louis, MO, USA). Initially, 3.3 × 10^5^ cells (0.5 mL) were seeded on 24-well plates for 20 h in the presence of 70 μM hemin to induce the intracellular production of ROS. Cells were then subjected to different concentrations of HU (100 and 200 μM) in the presence or absence of 70 μM hemin for 2 h. Next, the supernatants were discarded, the cell monolayers were gently washed twice with pre-heated (37°C) sterile saline (0.85% NaCl), followed by reincubation for 30 min with 10 μM of DCFH-DA probe in SFB-depleted medium without phenol red (Gibco, New York, NY, USA) to avoid probe degradation. Finally, the monolayers were washed twice with saline and trypsinized with 0.3 mL of trypsin-EDTA (0.25%) for 4 min at 37°C. Trypsin was neutralized with RPMI medium without phenol red supplemented with 10% SFB, and cells were transferred to sterile 1.5 mL microtubes, washed twice with saline solution and then placed in specific tubes for flow cytometry acquisition using Ex/Em: ~492–495/517–527 nm on a BD LSRFortessa™ cytometer (Biosciences, San Jose, CA, USA). ROS measurements are expressed by mean fluorescence intensity (MFI) and replicate values are expressed as means (10,000 events for each condition).

### Nitrite Accumulation in Supernatants

NO production was indirectly quantified in PBMC and HUVEC supernatants using the Griess method (21) after treatment with HU (100 and 200 μM) alone or in combination with 70 μM hemin for 24 h. First, 1.2 × 10^6^ PBMC/well (0.3 mL) and 8 × 10^4^ HUVEC/well (0.5 mL) were seeded on 48-well and 24 well-plates, respectively, in the presence of stimuli. Next, 50 μL (1:1, v/v) of the supernatant was added to Griess reagent [1% sulfanilamide and 0.1% naphthyl ethylenediamine dihydrochloride (Sigma Aldrich, St. Louis, MO, USA) in 2.5% H_3_PO_4_ solution] for 5 min. Absorbance was measured on a microplate reader at a wavelength of 550 nm. The conversion of absorbance into micromolar concentrations of NO was deduced from a standard curve using a known concentration of NaNO_2_ diluted in RPMI medium. The standard curves used to determine molar concentrations assumed a coefficient of determination (*R*^2^) value ≥ 0.999.

### Gene Expression and RNA Extraction Assays

HUVEC and PBMC were challenged with different HU concentrations in the presence and absence of 70 μM hemin for 4 h. Gene expression assays were performed by real-time quantitative reverse-transcription polymerase chain reaction (RT-qPCR). Total RNA was extracted from HUVEC and PBMC samples using TRIzol Reagent (Invitrogen, Carlsbad, CA, USA) according to the manufacturer's specifications. The concentration and purity of the extracted RNA were determined at the optical densities of 260 and 280 nm using a NanoDrop 2000 spectrophotometer (ThermoFisher Scientific, Rockford, IL, USA) at an absorbance ratio A_260/280_ of 1.90–2.02. Reverse cDNA synthesis by reverse transcription of RNA (RT-PCR) was performed using 250 ng of the RNA transcript in a High-Capacity cDNA Reverse Transcription Kit (ThermoFisher Scientific, Rockford, IL, USA) following the manufacturer's specifications. Real-time PCR was performed on an ABI PRISM 7500 Fast Real-Time PCR System (Applied Biosystems, Foster City, CA, USA) under the following cycling conditions: 95°C for 20 s, 95°C for 1 s, 60°C for 20 s for 40 cycles. For the RT-qPCR reactions, mixtures containing SYBR® Green PCR Master Mix (SYBR® Green I dye, AmpliTaq Gold® DNA Polymerase, dNTPs with dUTP, passive reference 1–ROX) (Applied Biosystems, Foster City, CA, USA), the primers specific to the target genes and 2 μL of the cDNA sample product were added to the optical plates. The primers used for quantitative PCR were as follows: [*HMOX1*: 5′-ATG GCC TCC CTG TAC CAC ATC-3′ (forward); 5′-TGT TGC GCT CAA TCT CCT CCT-3′ (reverse); *SOD1*: 5′-TGG CCG ATG TGT CTA TTG AA-3′ (forward); 5′-CAC CTT TGC CCA AGT CAT CT-3′ (reverse); *GSR*: 5′-ACT TGC CCA TCG ACT TTT TG-3′ (forward); 5′-GGT GGC TGA AGA CCA CAG TT-3′ (reverse); *GPX1*: 5′-CCA AGC TCA TCA CCT GGT CT-3′ (forward); 5′-TCG ATG TCA ATG GTC TGG AA-3′ (reverse); β-actin: 5′-CCT GGC ACC CAG CAC AAT-3′ (forward); 5′-GCC GAT CCA CAC GGA GTA CT-3′ (reverse); tubulin isotype a1C: 5′-TCA ACA CCT TCT TCA GTG AAA GG-3′ (forward); 5′-AGT GCC AGT GCG AAC TTC ATC (reverse). After determining the threshold cycle (CT), gene expression was measured by relative quantification using the following expression: fold-change = 2^−Δ(ΔCT)^, where ΔCT = CT_target_ – CT_housekeeping_ and Δ(ΔCT) = ΔCT_treated_ – ΔCT_control(medium)_. Beta-actin and tubulin isotype a1C were used as housekeeping genes.

### Microarray Assays with HUVEC

Microarray analyses were performed using a HumanHT-12 v.4 Expression BeadChip Kit (Illumina Inc., San Diego, CA, USA) and a TargetAmp™ Nano Labeling Kit for Illumina® Expression BeadChip® (Epicenter Technologies, Madison, Wisconsin, USA), in accordance with the manufacturers' specifications. Fluorescence values were acquired on an Illumina HiScan system using iScan Control software (Illumina Inc., San Diego, CA, USA). After quality control assessments, the generated data were exported for analysis using Genome Studio software (Illumina Inc., San Diego, CA, USA). Results with a detected *p* > 0.05 and a differential score <0.05 were discarded. After validation, the transcripts were selected and analyzed using Ingenuity Pathway Analysis (IPA) software (QIAGEN). Experiments were performed in triplicate and results reflect relative expression (log fold-change > 1.5), determined by comparing HUVECs treated with 200 μM HU to untreated cells.

### Statistical Analysis

Data are expressed as means ± standard deviation of at least one representative experiment. All experiments were performed in triplicate. One-way ANOVA followed by Tukey's *post-hoc* test was applied to test variance between multiple groups. Significance was considered when *p* < 0.05. GraphPad Prism software version v.6.0 was used for statistical analyses (GraphPad, San Diego, CA, USA).

## Results

### HU Scavenges Free Radicals

**Figure 1:**
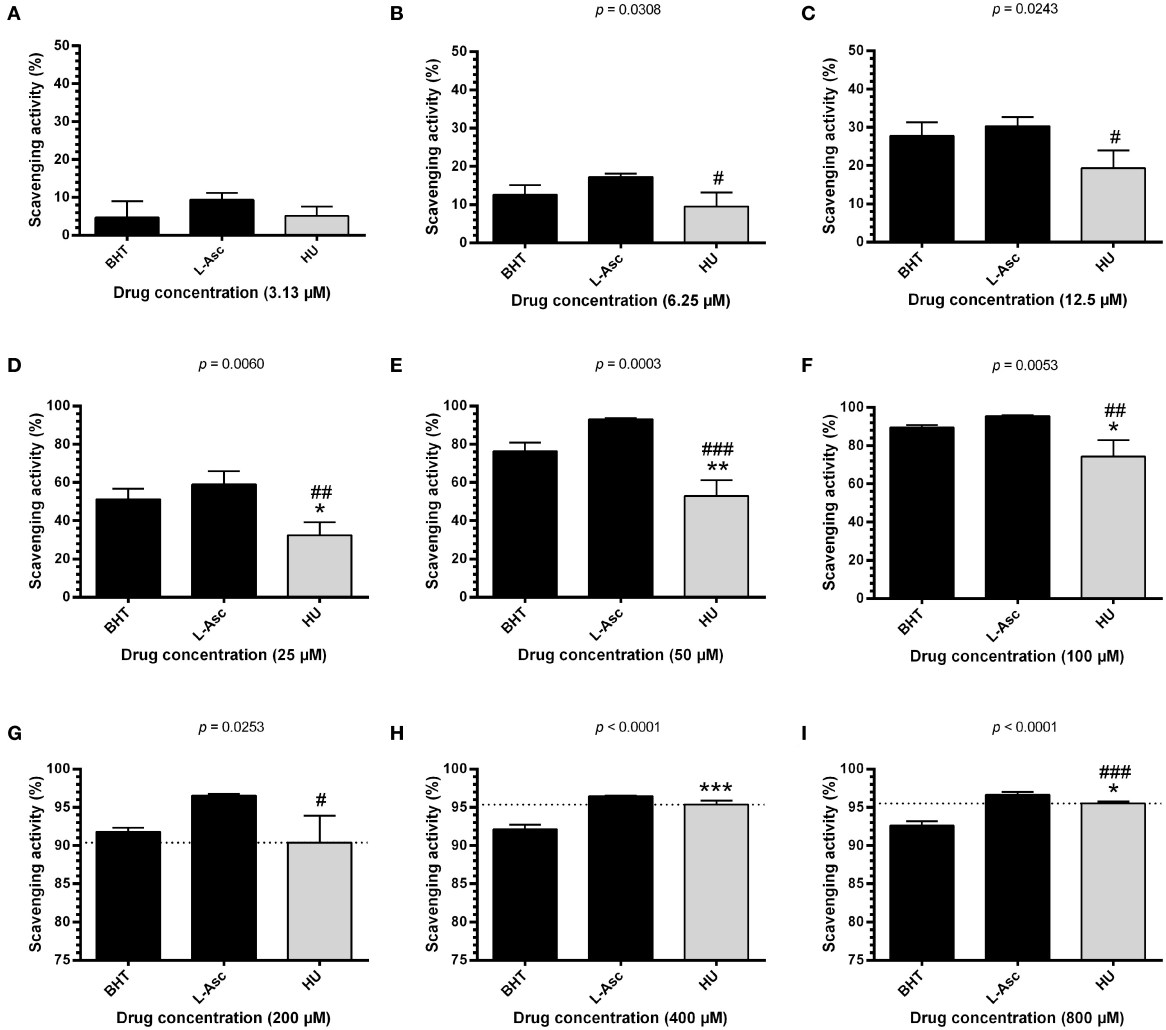
DPPH scavenging activity of different concentrations of hydroxyurea after 60 min of incubation. Antioxidant activity was measured by scavenging of the DPPH free radical using the HU concentrations 3.13 μM **(A)**, 6.25 μM **(B)**, 12.5 μM **(C)**, 25 μM **(D)**, 50 μM **(E)**, 100 μM **(F)**, 200 μM **(G)**, 400 μM **(H)** and 800 μM **(I)**. Results correspond to the mean ± standard deviation of four independent experiments. BHT and L-ascorbate were used as reference antioxidant compounds. HU, hydroxyurea; L-Asc, L-ascorbate; BHT, butylated hydroxytoluene. Statistical significance determined by one-way ANOVA, *p* < 0.0001, followed by Tukey's *post-hoc* test: HU vs. BHT: *p < 0.05, **p < 0.01, ***p < 0.001; HU vs. L-Asc, ^#^*p* < 0.05, ^*##*^*p* < 0.01, ^*###*^*p* < 0.001.

To investigate the possible antioxidant effects of HU, we performed assays evaluating radical scavenging activity using 100 μM DPPH, a stable free radical. Initially, we found that HU presents significantly superior scavenging activity at 60 min of incubation after standardization (Supplementary Figure 3). Next, radical scavenging assays involving DPPH demonstrated a concentration-dependent activity for HU. Our global analysis found that HU (*IC*_50_ = 38.68 ± 0.47 μM) presents lower DPPH radical scavenging activity than the reference antioxidant compounds BHT (*IC*_50_ = 23.07 ± 2.64 μM, *p* < 0.05) and L-ascorbate (*IC*_50_ = 18.22 ± 5.93 μM, *p* < 0.001) (Table 1). However, scavenging activity equivalent or superior to BHT was observed at concentrations ≥200 μM (Figure 1). Based on these findings, HU was used in all further assays at concentrations of 100 and 200 μM.

**Table 1:** Scavenging activity (corresponding to 50% of 100 μM DPPH) of hydroxyurea, L-ascorbate and butylated hydroxytoluene.

**Drugs**	**IC_**50**_ (μM) ± SD**	**One-way**	**Tukey's** ***post-hoc*** **test**		
		**ANOVA**			
			**HU vs. BHT**	**HU vs. L-Asc**	**L-Asc vs. BHT**
Hydroxyurea	38.68 ± 0.47	*p* < 0.0001	*p* < 0.0001	*p* < 0.0001	*p* < 0.05
Butylated hydroxytoluene	23.07 ± 2.64				
L-Ascorbate	18.22 ± 5.93				

*IC_50_ values correspond to the mean inhibitory concentration of three independent experiments. SD, Standard deviation; HU, Hydroxyurea; BHT, Butylated hydroxytoluene; L-Asc, L-Ascorbate*.

### HU and Hemin Present Non-Toxic Effects in HUVEC and PBMC

Cytotoxicity evaluations in HUVECs and PBMCs were carried out using resazurin sodium salt and propidium iodide methods, respectively. For toxicity testing in HUVECs, we initially standardized the time required to reduce resazurin sodium salt to a level equivalent to the same percentage of cell viability found in unstimulated cell cultures (Supplementary Figure 2A). No decreases in HUVEC viability were seen at the concentrations evaluated, ranging from 6.25 to 100 μM of hemin (Supplementary Figure 2B). Considering these findings, all following assays employed a hemin concentration of 70 μM, which corresponds to the plasmatic concentrations of free hemin observed in a previous study by our group involving steady-state SCA patients. No toxicity was observed in the HUVEC and PBMC samples at any of the HU or hemin concentrations evaluated (Supplementary Figures 2C,D).

### HU Increases NO Production and Decreases the Formation of Cytosolic ROS in HUVEC Treated with HU plus Hemin

**Figure 2:**
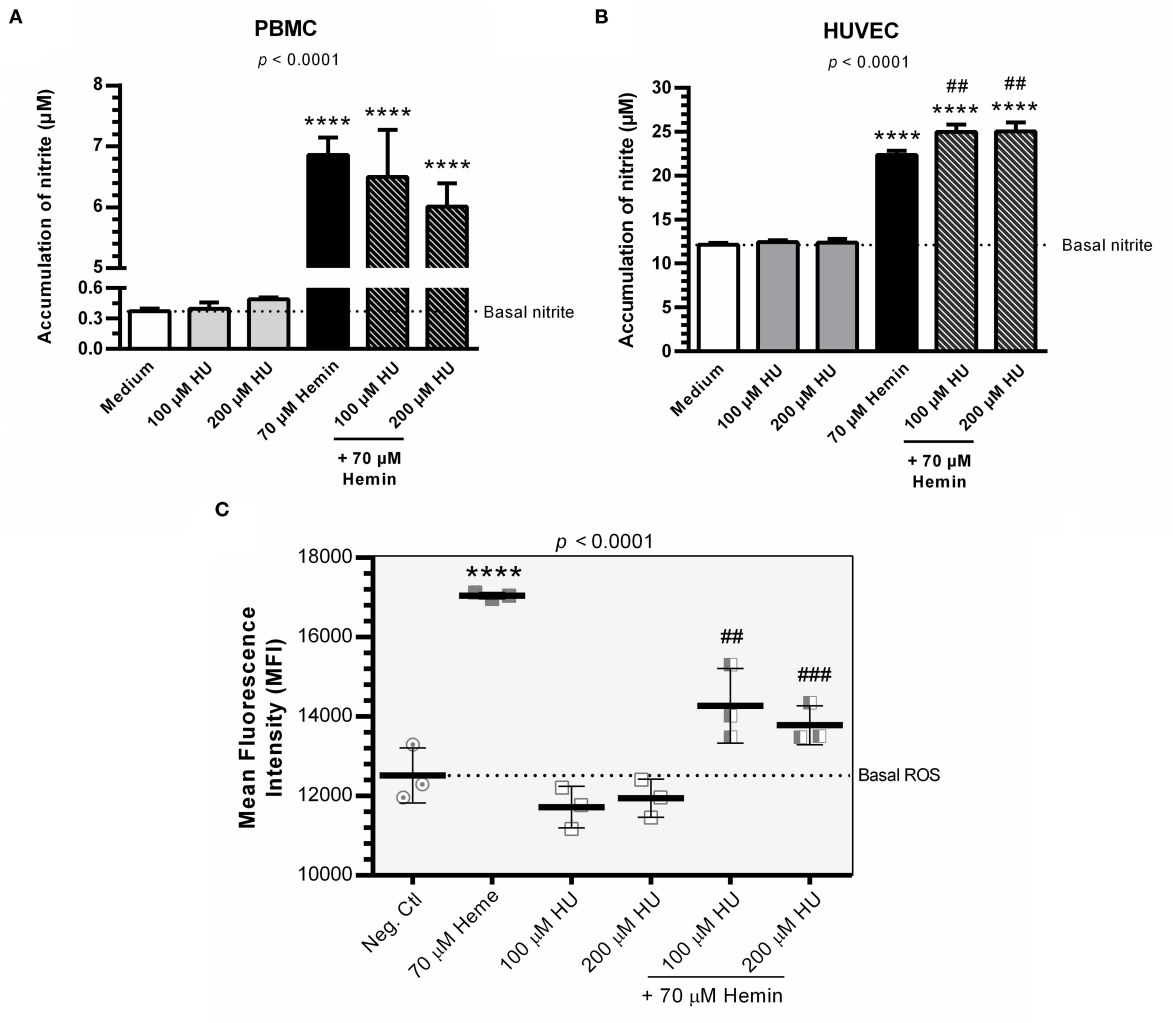
NO production and formation of intracellular ROS in the presence of Hydroxyurea and/or hemin. **(A)** Production of NO in supernatants of PBMCs in response to various treatment protocols. Results correspond to means ± SD of three independent experiments. Statistical significance determined by one-way ANOVA, *p* < 0.0004, followed by Tukey's *post-hoc* test: HU alone or in combination with hemin vs medium, or hemin vs medium, **p < 0.01; HU + hemin vs hemin, ^*##*^*p* < 0.01. **(B)** Production of NO in supernatants of HUVECs in response to various treatment protocols. Results correspond to the mean ± SD of three independent experiments. Statistical significance determined by one-way ANOVA, *p* < 0.0001, followed Tukey's *post-hoc* test: HU alone or HU + hemin vs. medium, or hemin vs. medium, ****p < 0.0001; HU + hemin vs. hemin, ^*##*^*p* < 0.01. **(C)** Decreased ROS formation in HUVECs using the oxidant-sensing fluorescent probe 10 μM 2', 7'-dichlorodihydrofluorescein diacetate (DCFH-DA). Statistical significance determined by mean fluorescence intensity (MFI) values representative of the mean ± SD of three experimental replicates. One-way ANOVA, *p* < 0.0001, followed by Tukey's *post-hoc* test: HU alone or associated with hemin vs. medium, or hemin vs. medium, ****p < 0.0001; HU + hemin vs. hemin, ^*##*^*p* < 0.01; ^*###*^*p* < 0.001.

Hemin alone was shown to induce NO production in PBMCs and HUVECs. PBMCs and HUVECs treated with HU at 100 μM and 200 μM did not show any significant increases in NO (Figures 2A,B). However, when we evaluated the combined treatment of HU plus hemin vs. negative controls or hemin alone, significantly increased NO production was seen only in HUVECs. HU plus 70 μM hemin was found to markedly reduce ROS in HUVECs in a concentration dependent-manner (Figure 2C).

### Treatments with HU Alone or Combined with Hemin Induce Antioxidant Enzyme Gene Expression in HUVEC and PBMC

**Figure 3:**
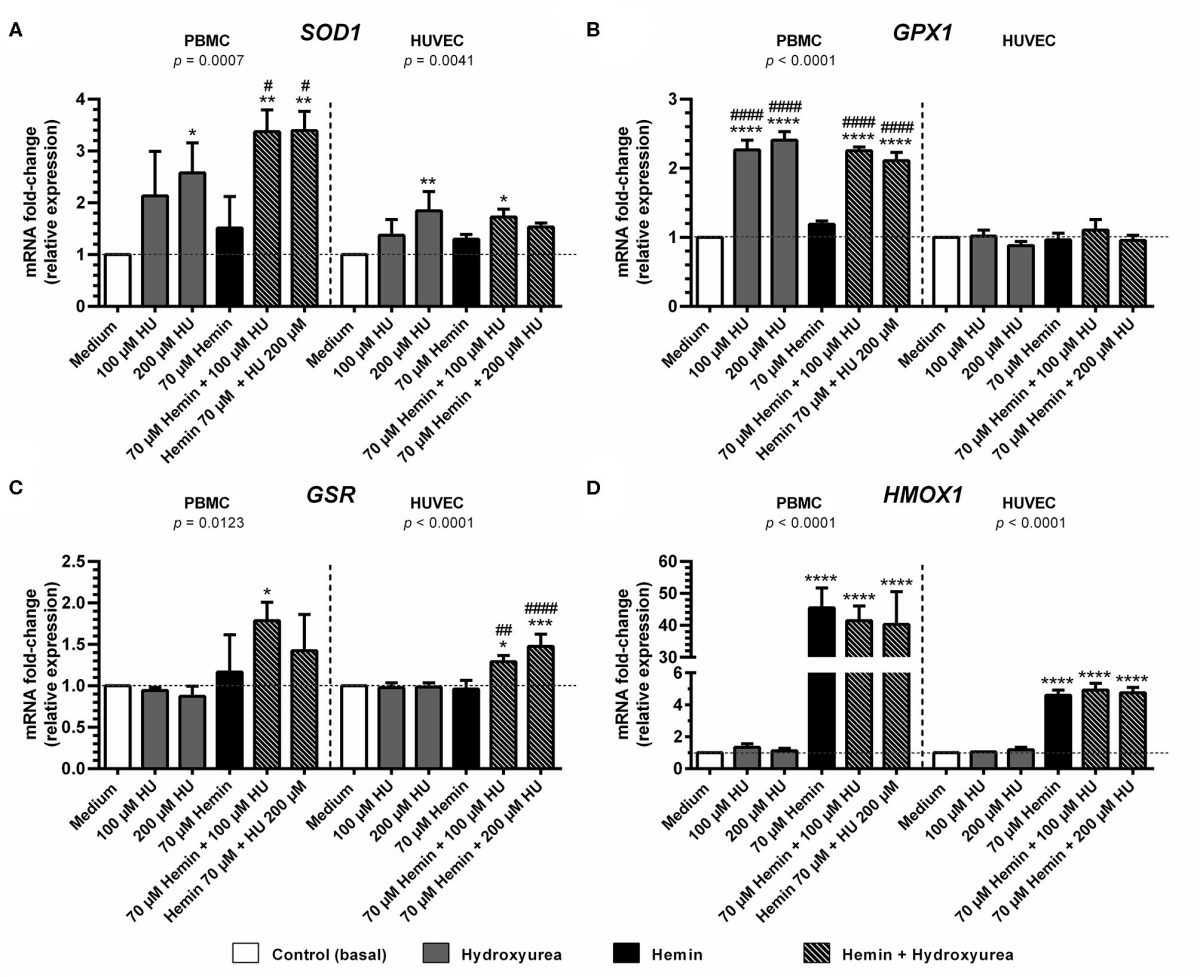
Effect of Hydroxyurea on induction of antioxidant response gene expression in PBMC and HUVEC treated with different concentrations of hydroxyurea (100 and 200 μM) in the presence or absence of 70 μM hemin for 4 h. **(A)** superoxide dismutase-1 (*SOD1*); **(B)** glutathione peroxidase (*GPX1*); **(C)** glutathione-disulfide reductase (*GSR*); **(D)** Heme-oxygenase 1 (*HMOX1*). Results correspond to the mean ± standard deviation of three independent experiments. Expression values determined by relative quantification using the following expression: fold-change = 2^−Δ(ΔCT)^, where ΔCT = CT_target_-CT_housekeeping_ and Δ(ΔCT) = ΔCT_treated_−ΔCT_control_ (medium). Data were normalized to represent fold expression above controls for each gene. Statistical significance determined by one-way ANOVA, *p* < 0.05, followed by Tukey's *post-hoc* test: HU alone or HU + hemin vs. medium, or hemin vs. medium, *p < 0.05; **p < 0.01, ***p < 0.001, ****p < 0.0001; hemin + HU vs. hemin, ^#^*p* < 0.05; ^*##*^*p* < 0.01; ^*####*^*p* < 0.0001.

Treatment with 200 μM HU increased the expression of *SOD1* in PBMCs and HUVECs by 2.57 ± 0.86-fold (*p* < 0.05) and 1.84 ± 0.36-fold, (*p* < 0.01), respectively, compared to negative controls (Figure 3A). Combined treatments using 100 and 200 μM of HU plus 70 μM hemin promoted a statistically significant increase of 3.37 ± 0.42-fold (*p* < 0.01) and 3.39 ± 0.37-fold (*p* < 0.01) in *SOD1* expression in PMBCs, vs. 1.53 ± 0.07-fold (*p* < 0.05) in HUVECs (100 μM HU plus hemin).

Considerable *GPX* expression was observed in PBMCs treated with 100 μM (2.27 ± 0.14-fold, *p* < 0.0001) and 200 μM HU (2.40 ± 0.12-fold; *p* < 0.0001) (Figure 3B). Similar expression values were observed in hemin-treated PBMCs at both HU concentrations (2.25 ± 0.05-fold, *p* < 0.001 and 2.11 ± 0.11-fold, respectively). In contrast, *GPX* expression levels in HUVECS did not vary in response to the treatments.

Treatment with HU alone did not provoke increased *GSR* expression at any of the concentrations evaluated in either cell type evaluated (Figure 3C). However, an increase in *GSR* expression was observed in PBMCs and HUVECs submitted to combined HU plus hemin treatment. PBMCs treated with 100 μM or 200 μM of HU and 70 μM of hemin presented 1.79 ± 0.22-fold (*p* < 0.05) and 1.42 ± 0.43-fold increases in *GSR* expression, respectively, while HUVECs presented 1.30 ± 0.07 (*p* < 0.05) and 1.48 ± 0.15-fold (*p* < 0.001) higher expression in comparison to the negative control.

Significantly higher levels of *HMOX1* were observed in hemin-treated PBMCs and HUVECs, regardless of HU concentration (Figure 3D). In PBMCs, increased *HMOX1* expression was 45.5 ± 6.2-fold (*p* < 0.0001) vs. controls. HUVECs exhibited modest increases in *HMOX1* expression (4.6 ± 0.32-fold; *p* < 0.0001) compared to PBMCs, despite high statistical significance. Despite a slight decrease in *HMOX1* expression in PBMCs treated with 100 μM (41.5 ± 4.6-fold) and 200 μM HU (40.31 ± 10.2-fold) compared to hemin alone, combined HU plus hemin treatment did not significantly reduce this expression.

### Microarray Analysis in HUVEC Suggests that HU Induces the Nrf2-Antioxidant Response Element/Electrophile Signaling Pathway Regulated by p62/SQSTM1

Preliminary canonical pathway analysis identified 39 genes related to Nrf2-mediated oxidative stress response in HUVECs (Table 2). HU treatment induced increased expression levels of *SOD2* (1.852 Expr Log Ratio), *GSR* (2.882 Expr Log Ratio), *GSTM2* (2.210 Expr Log Ratio), microsomal glutathione S-transferase 1 (*MGST1*) (1.733 Expr Log Ratio) and carbonyl reductase 1 (*CR1*) (1.727 Expr Log Ratio). We also found increased expression of phosphatidylinositol-4-phosphate 3-kinase catalytic subunit type 2 beta (*PIK3C2B*) (1.892 Expr Log Ratio), phosphoinositide-3-kinase regulatory subunit 3 (*PIK3R*) (1.597 Expr Log Ratio), protein kinases C beta (*PRKCB*) (4.026 Expr Log Ratio) and zeta (*PRKCZ*) (1.902 Expr Log Ratio), and glycogen synthase kinase 3 beta (*GSK3B*) (1.607 Expr Log Ratio). Moreover, HU induced increased p62/sequestosome (*p62/SQSTM1*) (1.639 Expr Log Ratio) expression and decreased expression of BTB domain and CNC homolog 1 (*BACH1*) (-1.721 Expr Log Ratio), as well as ubiquitin-conjugating enzyme E2 K (*UBE2K*) (−1.830 Expr Log Ratio). Table 3 presents the results of our upstream analyses, which predicted the activation of mature microRNAs, such as miR-155-5p (activation z-score = 2.840) and miR-141-3p (activation z-score = 2.801), as well as the activation of the Jun transcription regulator (activation z-score = 2.560).

**Table 2:** Differential expression of genes involved in Nrf2-antioxidant/electrophile response element signaling pathway, identified through microarray analysis of HUVEC treated with hydroxyurea.

**Symbol**	**Gene name**	**Expr log ratio^‡^**	**Location**	**Type**
**ENZYMES**				
*GSR*	Glutathione-disulfide reductase	2.882	Cytoplasm	Enzyme
*GSTM2*	Glutathione S-transferase mu 2	2.210	Cytoplasm	Enzyme
*KLB*	Klotho beta	1.974	Plasma membrane	Enzyme
*SOD2*	Superoxide dismutase 2	1.852	Cytoplasm	Enzyme
*HACD3*	3-hydroxyacyl-CoA dehydratase 3	1.807	Cytoplasm	Enzyme
*MGST1*	Microsomal glutathione S-transferase	1.733	Cytoplasm	Enzyme
*CBR1*	Carbonyl reductase 1	1.727	Cytoplasm	Enzyme
*RRAS*	RAS related	1.517	Cytoplasm	Enzyme
*NRAS*	NRAS proto-oncogene, GTPase	−1.521	Plasma Membrane	Enzyme
*AOX1*	Aldehyde oxidase 1	−2.188	Cytoplasm	Enzyme
**PEPTIDASES**				
*CLPP*	Caseinolytic mitochondrial matrix peptidase proteolytic subunit	1.551	Cytoplasm	Peptidase
*ENC1*	Ectodermal-neural cortex 1	−1.996	Nucleus	Peptidase
*EPHX1*	Epoxide hydrolase 1	−3.291	Cytoplasm	Peptidase
**TRANSCRIPTION REGULATOR**				
*SQSTM1*	Sequestosome 1	1.639	Cytoplasm	Transcription regulator
*ATF4*	Activating transcription factor 4	−1.639	Nucleus	Transcription regulator
*BACH1*	BTB domain and CNC homolog 1	−1.721	Nucleus	Transcription regulator
*PMF1/PMF1-BGLAP*	Polyamine modulated factor 1	−1.740	Nucleus	Transcription regulator
*CREBBP*	CREB binding protein	−1.743	Nucleus	Transcription regulator
*MAFG*	MAF bZIP transcription factor G	−1.823	Nucleus	Transcription regulator
*UBE2K*	Ubiquitin conjugating enzyme E2 K	−1.830	Cytoplasm	Transcription regulator
*FOS*	Fos proto-oncogene, AP-1 Transcription factor subunit	−3.950	Nucleus	Transcription regulator
**KINASE/OTHERS**				
*PRKCB*	Protein kinase C beta	4.026	Cytoplasm	Kinase
*PRKCZ*	Protein kinase C zeta	1.902	Cytoplasm	Kinase
*PIK3C2B*	Phosphatidylinositol-4-phosphate 3-kinase catalytic subunit type 2 beta	1.892	Cytoplasm	Kinase
*DNAJB12*	DnaJ heat shock protein family (Hsp40) member B12	1.794	Cytoplasm	Other
*FGFR3*	Fibroblast growth factor receptor 3	1.685	Plasma Membrane	Kinase
*GSK3B*	Glycogen synthase kinase 3 beta	1.607	Nucleus	Kinase
*PIK3R3*	Phosphoinositide-3-kinase regulatory subunit 3	1.597	Cytoplasm	Kinase
*PIK3C2A*	Phosphatidylinositol-4-phosphate 3-kinase catalytic subunit type 2 alpha	−1.716	Cytoplasm	Kinase
*PIK3CB*	Phosphatidylinositol-4,5-bisphosphate 3-kinase catalytic subunit beta	−1.761	Cytoplasm	Kinase
*DNAJB14*	DnaJ heat shock protein family (Hsp40) member B14	−1.532	Cytoplasm	Enzyme
*DNAJC21*	DnaJ heat shock protein family (Hsp40) member C21	−1.649	Other	Other
*FRS2*	Fibroblast growth factor receptor substrate 2	−1.665	Plasma Membrane	Kinase
*PIK3R1*	Phosphoinositide-3-kinase regulatory subunit 1	−1.690	Cytoplasm	Kinase
*PRKCE*	Protein kinase C epsilon	−1.679	Cytoplasm	Kinase
*DNAJB4*	DnaJ heat shock protein family (Hsp40) member B4	−1.843	Nucleus	Other
*DNAJC18*	DnaJ heat shock protein family (Hsp40) member C18	−1.897	Other	Enzyme
*GAB1*	GRB2 associated binding protein 1	−2.156	Cytoplasm	Kinase
*MAPK14*	Mitogen-activated protein kinase 14	−2.418	Cytoplasm	Kinase

‡ *Based on relative expression (log fold-change > 1.5)*.

**Table 3:** Upstream analysis of genes identified through microarray analysis of HUVEC treated with hydroxyurea.

**Upstream regulator**	**Molecule type**	**Predicted activation state**	**Activation z-sore**	***p*-value of overlap**
15-deoxy-delta-12,14 -PGJ 2	Chemical–endogenous non-mammalian	Inhibited	−2.125	1.00E00
Pkc(s)	Group	Inhibited	−3.043	1.00E00
Vegf	Group	Inhibited	−2.126	1.41E−02
PRKAA2	Kinase	Inhibited	−2.101	4.06E−03
CD24	Other	Inhibited	−4.459	5.89E−04
GJA1	Transporter	Inhibited	−2.190	2.56E−02
FOXM1	Transcription regulator	Inhibited	−2.242	1.40E−06
FOXO1	Transcription regulator	Inhibited	−2.628	2.12E−01
S100A6	Transporter	Inhibited	−2.345	1.42E−02
YAP1	Transcription regulator	Inhibited	−2.449	1.23E−02
TCF4	Transcription regulator	Inhibited	−2.252	2.59E−02
OSM	Cytokine	Inhibited	−2.123	4.56E−01
ESR1	Ligand-dependent nuclear receptor	Inhibited	−2.662	3.28E−07
Ellagic acid	Chemical–endogenous non-mammalian	Inhibited	−2.000	4.71E−02
Imatinib	Chemical drug	Inhibited	−2.097	3.11E−01
GW9662	Chemical reagent	Inhibited	−2.055	2.31E−01
Isoproterenol	Chemical drug	Inhibited	−2.789	3.52E−01
Cholecalciferol	Chemical–endogenous mammalian	Inhibited	−2.331	1.00E00
R-WIN 55,212	Chemical reagent	Inhibited	−2.063	3.88E-04
zVAD-FMK	Chemical–protease inhibitor	Inhibited	−2.000	1.36E-01
Cocaine	Chemical drug	Inhibited	−2.193	1.00E00
25-hydroxycholesterol	Chemical reagent	Inhibited	−2.190	1.00E00
Hyaluronic acid	Chemical–endogenous mammalian	Activated	2.000	1.00E00
E2f	Group	Activated	2.725	8.25E−06
SPDEF	Transcription regulator	Activated	2.158	2.85E−01
EPAS1	Transcription regulator	Activated	2.059	1.00E00
SPI1	Transcription regulator	Activated	2.565	1.00E00
miR-155-5p (miRNAs w/seed UAAUGCU)	Mature microRNA	Activated	2.840	2.12E−01
mir-15	MicroRNA	Activated	2.277	9.16E−02
miR-29b-3p (and other miRNAs w/seed AGCACCA)	Mature microRNA	Activated	2.255	4.19E−01
miR-141-3p (and other miRNAs w/seed AACACUG)	Mature microRNA	Activated	2.801	2.69E−02
mir-145	MicroRNA	Activated	2.236	4.67E−01
NUPR1	Transcription regulator	Activated	4.357	1.63E−05
JUN	Transcription regulator	Activated	2.560	1.00E00
SRSF3	Other	Activated	2.229	1.78E−02
KLF4	transcription regulator	Activated	2.020	1.00E00
SYK	Kinase	Activated	2.695	1.78E−01
TBX5	Transcription regulator	Activated	2.000	1.00E00
MEOX2	Transcription regulator	Activated	2.200	4.96E−01
IFNB1	Cytokine	Activated	2.183	1.00E00
IL15	Cytokine	Activated	2.280	1.00E00
Sulindac sulfide	Chemical drug	Activated	2.192	2.65E−01
GW3965	Chemical reagent	Activated	2.204	1.00E00
Mifepristone	Chemical drug	Activated	2.201	3.74E−01

## Discussion

The present study aimed to confirm the antioxidant potential of HU and investigate its effects on the modulation of the antioxidant cellular response. DPPH scavenging activity assays revealed that, despite higher *IC*_50_ values determined for HU at 100 and 200 μM, HU demonstrated considerable scavenging activity compared to controls. This finding suggests that HU may be able to directly neutralize free radicals in the extracellular microenvironment, which could be explained by its ability to donate a hydrogen atom electron in the neutralization of the radical compound DPPH (22). Moreover, our results also indicate that HU may scavenge ROS/RNS by inducing the antioxidant enzyme system. This finding is of great importance, as HU could potentially confer an important protective effect against direct oxidative attacks on membrane phospholipids, as well as prevent/minimize the triggering of activation responses involved in the initiation of the oxidative cascade and establishment of inflammation (23–26).

Both concentrations mentioned above are consistent with the plasma levels of HU generally observed in patients with SCA, which have been extensively used in *in vitro* studies (27–30). Accordingly, we chose these concentrations for our additional assays, in addition to combined therapy with hemin at 70 μM based on the findings from Carvalho et al. (31). Cytotoxicity assays involving hemin did not reveal any significant effects on cell viability in either PBMCs or HUVECs. This may be explained by the degree of resilience both cell types present in the pro-oxidative microenvironment promoted by hemin. It was previously shown that hemin can induce HO-1 production in monocytes, which promotes a cytoprotective effect through the inhibition of apoptosis (32). Our results corroborate this finding, as we observed higher levels of *HMOX1* expression in PBMCs and HUVECs following treatment with hemin. HUVECs treated with HUs plus hemin presented significant increases in NO production, which corroborates previously published results. Other studies have suggested that treatment with HU in the presence of heme resulted in the production of iron nitrosyl-heme (Fe^2+^-NO), nitrite, and nitrate in SCA individuals (33–37).

Our investigation of antioxidant gene expression indicated differential expression profiles for each cell type after 4 h of incubation with HU in combination or not with hemin. Higher gene expression was seen in PBMCs than in HUVECs, which can be explained by the substantial capacity of recognition and effector responses in leukocytes, especially monocytes, present in PBMCs (38, 39). Our results show that treatment with HU in combination or not with hemin significantly provoked increases in *SOD1* and *GSR* expression in both cell types, similarly to the higher *GPX* expression found in PBMCs. Previous studies have demonstrated that HU activates the *GPX*-mediated NO-cGMP pathway in patients with SCA (40, 41). This activation may be due to the induction of transcriptional factors and/or H_2_O_2_ production controlled by the production of GPX, which is dependent on the reduced glutathione (GSH) synthesized by GSR (33, 42–45). This would seem to corroborate the higher levels of *SOD1, GPX* and *GSR* expression found herein in response to HU treatment. Interestingly, no association between HU and *HMOX1* expression was found, suggesting that the mechanism by which the antioxidant response system becomes activated does not involve the activation of *HMOX1*.

Microarray analyses were performed in HUVECs treated with HU to investigate the possible pathways involved in the antioxidant response system. HU induced significant increases in the expression of genes encoding antioxidant enzymes, such as *SOD2, GSR, GSTM2, CBR1, MGST1*, and *KLB*, as well as *p62/SQSTM1*. This antisickling agent was also associated with decreases in *BACH1* and *UBE2K* expression. Studies have demonstrated a positive correlation between *p62/SQSTM1* expression and Nrf2 induction (46–48), leading to the activation of antioxidant systems (49–51). BACH1 acts as a negative regulator of Nrf2, preventing the induction of an antioxidant response, while UBE2K is involved in Nrf2 degradation via the ubiquitin-proteasome system (49, 52, 53). Accordingly, the negative correlations observed between *p62/SQSTM1* and *BACH1*, as well as between *p62/SQSTM1* and *UBE2K*, suggest that HU may be capable of inducing an antioxidant response via the Nrf2 signaling pathway.

HUVECs treated with HU also presented increased expression of genes encoding *PIK3C2B, PIK3R3, PRKCB, PRKCZ* and *GSK3B*. Previous results have demonstrated that the activation of these genes is associated with the induction of the antioxidant response, mediated by the Nrf2 signaling pathway (54–57).

In addition, our upstream analyses performed in HUVECs treated with HU indicate the activation of miR-155-5p and miR-141-3p, which are involved in the inhibition of *BACH1* and *Keap1*, respectively, in addition to the activation of Jun, which is involved in the activation of the Nrf2-mediated antioxidant pathway (55, 58–60).

Our results suggest that HU directly scavenges free radicals and can induce the expression of antioxidant genes via induction of the Nrf2 signaling pathway. In addition, the findings herein preliminarily expand on the previously described primary mechanisms of HU, i.e., the induction of HbF production and NO release. However, further *in vitro* and *in vivo* studies will be necessary to validate the role of the Nrf2-mediated antioxidant pathway proposed by the present study.

## Data Availability Statement

All datasets generated for this study are included in the article/Supplementary Material.

## Ethics Statement

The present study received approval from the Institutional Review Board of the Gonçalo Moniz Institute of the Oswaldo Cruz Foundation (IGM-FIOCRUZ). The patients/participants provided their written informed consent to participate in this study.

## Author Contributions

SS, TP, and MG conceived and designed the study, performed statistical analyses, and wrote the manuscript. SS performed all experiments. JS, DZ, JV, SY, CA, SV, NL, and VB assisted in some experimentation and provided technical support and discussed the results and participated in manuscript elaboration. MG, TP, and SY critically revised the manuscript. All authors revised and approved the final version of the manuscript.

## Conflict of Interest

The authors declare that the research was conducted in the absence of any commercial or financial relationships that could be construed as a potential conflict of interest.

## Abbreviations

BACH1: BTB (Broad-Complex, Tramtrack and Bric-a-brac) Domain and CNC Homolog 1 Basic Leucine Zipper Transcription Factor 1
CBR1: Carbonyl reductase 1
DPPH, 2: 2-Diphenyl-1-picrylhydrazyl
GPX: Glutathione peroxidase
GSH: Reduced glutathione
GSR: Glutathione-disulfide reductase
GST: Glutathione S-transferase
GSTM2: Glutathione S-transferase mu 2
H_2_O_2_: Hydrogen peroxide
HbF: Fetal hemoglobin
HbS: Hemoglobin S
*HMOX1*: Heme oxigenase-1 gene
HU: Hydroxyurea
HUVEC: Human umbilical vein endothelial cells
Keap1: Kelch-like ECH-associated protein1
KLB: Klotho beta
MAPK: Mitogen-activated protein kinase
MGST1: Microsomal glutathione S-transferase
${\text{NO}}_{3}^{-}$: Nitrate
Nrf2: Nuclear factor erythroid 2 (NF-E2) p45-related factor 2
NO: Nitric oxide
PBMC: Peripheral blood mononuclear cells
p62/SQSTM1: Sequestosome1
RNS: Reactive nitrogen species
ROS: Reactive oxygen stress
SCA: Sickle cell anemia
SOD-1: Superoxide dismutase-1.
