# Supplementary material for: Hydroxyurea Scavenges Free Radicals and Induces the Expression of Antioxidant Genes in Human Cell Cultures Treated With Hemin
Source: Front Immunol. 2020 Jul 17;11:1488. doi: 10.3389/fimmu.2020.01488 (PMC7380266; doi:10.3389/fimmu.2020.01488)
Supplement: Supplementary file 1 [file Data_Sheet_1.DOCX]

**
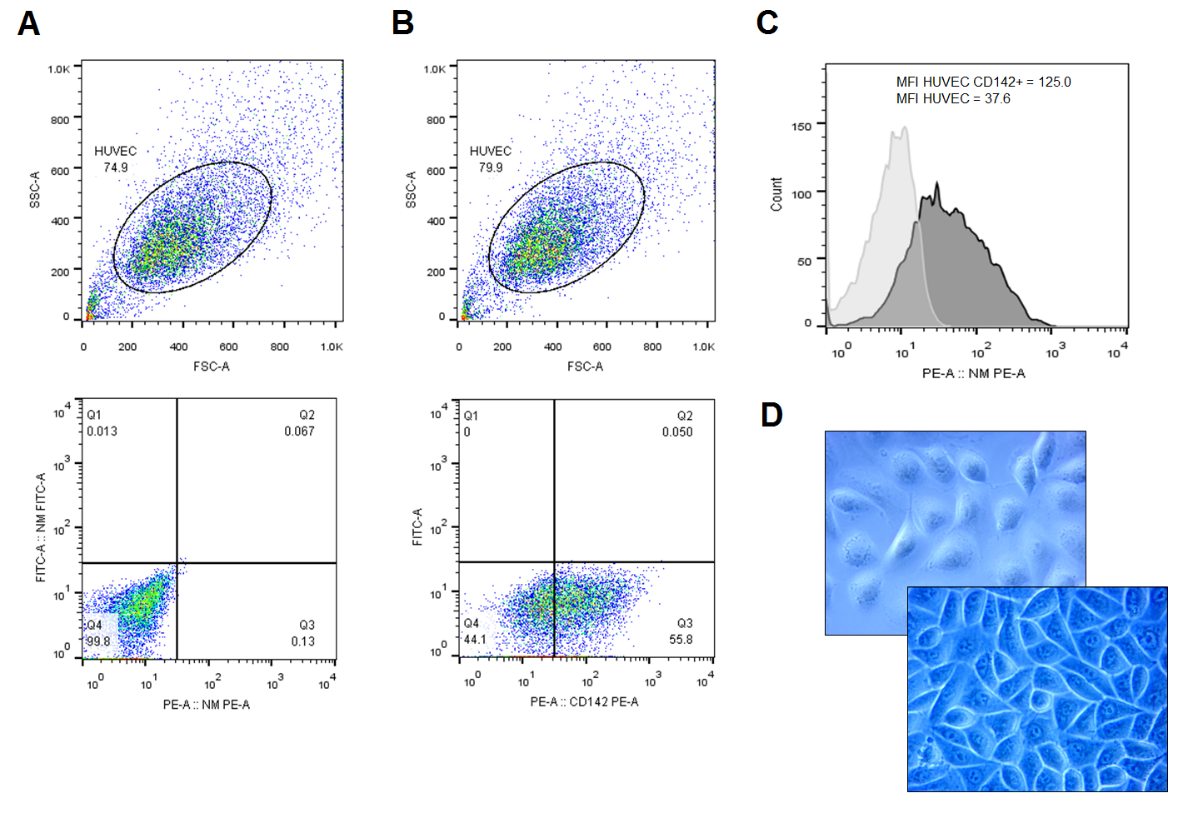
SUPPLEMENTARY DATA**

**Supplementary figure 1.** Phenotypic and morphological characterization of HUVEC by flow cytometry and light microscopy, detailing gating strategy and dot-plots, respectively. **(A)** HUVEC untreated (unlabeled) and **(B)** labeled (anti-CD142^+^, tissue factor) after pre-stimulation (2 h) with 200 μM H_2_O_2_. **(C)** Mean Fluorescence Intensity (MFI) of unlabeled HUVEC = 37.6; and MFI CD142^+^ HUVEC = 125.0. **(D)** Photomicrographs illustrate the typical cobblestone morphology of the oval nucleus and monolayer growth of the HUVEC cell line used in the assays under light microscopy (magnification 40x). Newly adhered cells (shown above) at the beginning of confluence. Confluent cells (below) detailing monolayer formation.

**
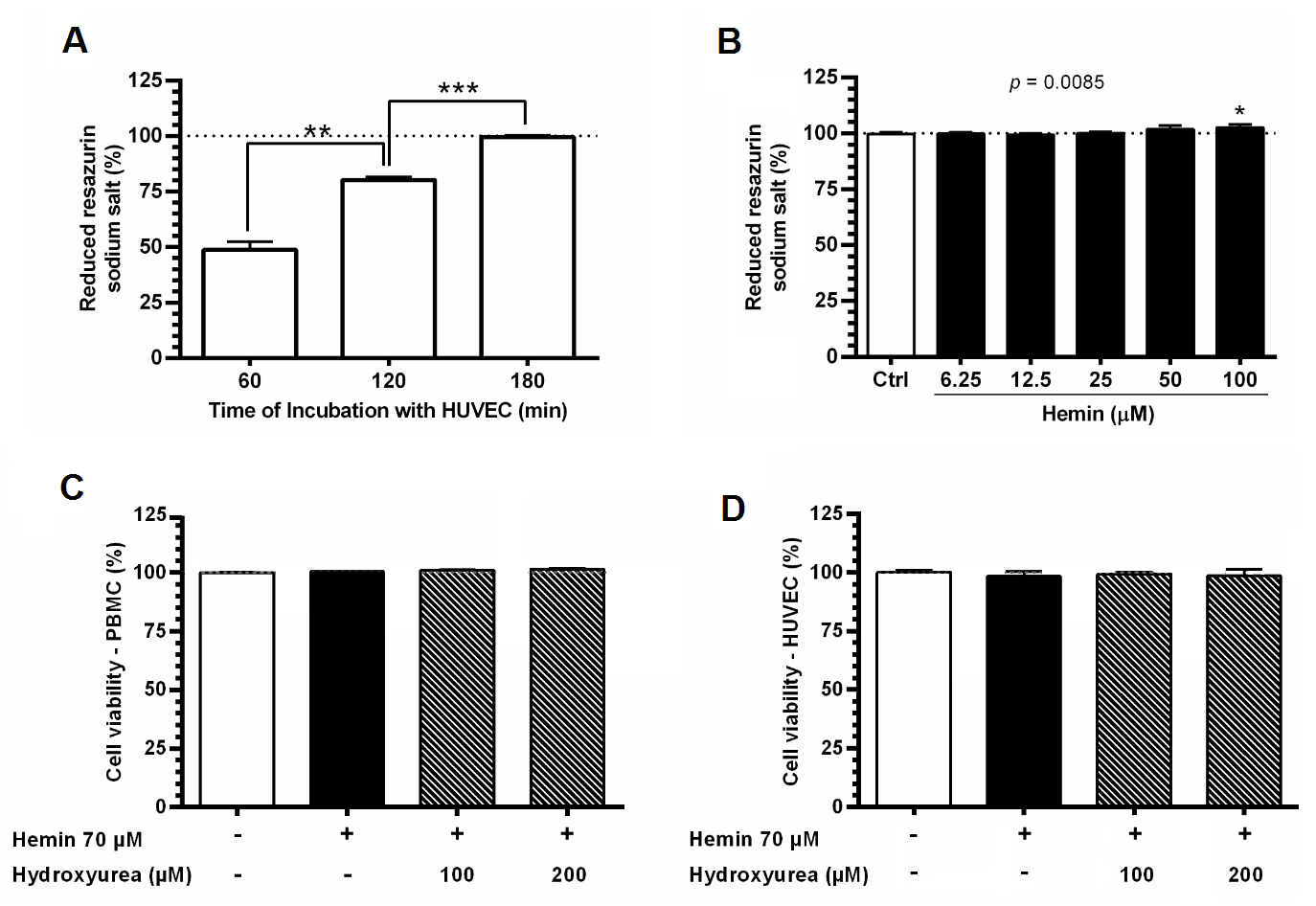
**

**supplementary figure 2.** Evaluation of hemin and hydroxyurea cytotoxicity in PBMCs e HUVECs. **(A)** Kinetic standardization of 12.5 µM resazurin sodium salt reduction and cytotoxicity in HUVECs at different hemin concentrations after 60, 120 and 180 min. Wilcoxon test, ***p* < 0.01. **(B)** Determination of hemin cytotoxicity at concentrations ranged between 6.25 and 100 μM after 24 h of treatment. Statistical significance determined by one-way ANOVA, *p* = 0.0085, followed by Tukey’s post-hoc test: Hemin *vs* positive viability control (medium), **p* < 0.05. **(C)** Cytotoxicity tests in PBMC treated with 70 µM hemin and hydroxyurea using propidium iodide. **(D)** HUVEC treated with 70 µM hemin and hydroxyurea using resazurin sodium salt (12.5 µM). Results correspond to the mean ± standard deviation of three independent experiments.

**

**

**Supplementary figure 3.** Scavenging activity (corresponding to 50% of 100 μM DPPH) of hydroxyurea, L-ascorbate and butylated Hydroxytoluene after 30 and 60 min of incubation. Results correspond to the mean ± standard deviation of three independent experiments. BHT and L-acorbate were used as reference antioxidant drugs. HU: hydroxyurea; L-Asc: L-ascorbate; BHT: butylated hydroxytoluene. Paired *t*-test, ***p* < 0.01.
